# Electrocautery-Assisted Management of Aberrant Frena Causing Midline Diastema: A Case Report

**DOI:** 10.7759/cureus.64321

**Published:** 2024-07-11

**Authors:** Lavannya Phaye, Unnati Shirbhate, Pavan Bajaj

**Affiliations:** 1 Periodontics, Sharad Pawar Dental College, Datta Meghe Institute of Higher Education and Research, Wardha, IND

**Keywords:** aesthetic, midline diastema, hemostasis, conventional, abberant frenum, lasers, frenectomy, electrocautery

## Abstract

Various oral complications such as gingival recession, restricted lip movement and tooth malalignment are the result of an abnormal frenum. Management of these types of frenum is either frenectomy or frenotomy. Methods for performing frenectomies include the conventional scalpel technique, Z-plasty, Miller's technique, V-Y plasty, lasers, and electrocautery. This case report details the successful management of an abnormal frenum attachment using electrocautery to ease and reduce discomfort to the 19-year-old female patient, causing aesthetic concerns. For its precision, minimal bleeding and post-operative discomfort, electrocautery was chosen. This procedure was performed under local anaesthesia. There were favourable post-operative outcomes as the patient experienced minimal pain and rapid recovery from the surgical site. Significant improvement in gingival health was seen in the follow-up examination. This case demonstrates the efficacy of using electrocautery in managing abnormal frenum attachment while highlighting its benefits over traditional surgical methods for ease and reduced discomfort.

## Introduction

The median maxillary labial frenum (MMLF), or Sewerins’s frenum, is a fibrous tissue band connecting to the bones (mandible, maxilla) above the muscle attachment [1]. The presence of an abnormal frenum is a critical factor in contributing to the persistence of a midline diastema, making it more evident for clinicians to pay more attention to the abnormal frenum attachment. Abnormal frenum attachment can also cause “gingival recession” if it is punctiliously attached to the gingival margin. Other causes of gingival recession might be the opening of the gingival crevice, muscle pull, or improper toothbrush placement, sometimes resulting in periodontal problems and tooth loss [2].

Frenal attachment was classified based on anatomic attachment site as clinical and morphological. Frenum attachment was categorised into four types: mucosal when the mucogingival junction is connected to the frenal fibers, gingival when the fibers are placed within the attached gingiva and when the fibers enter the interdental papilla, they are called papillary. When the frenal fibers extend to the palatine papilla from the alveolar process, this is known as papilla penetration [3]. The two surgical procedures are used to correct such issues: frenectomy and frenotomy. In frenotomy, an incision is made, and the frenal attachment is moved, while in frenectomy, along with attachment to the underlying bone, the frenum is removed [4]. The surgical removal of the aberrant frenum is mostly carried out by conventional scalpel technique but newer modalities such as electrocautery and diode laser are advantageous over the conventional in terms of ease of performing surgery, dental anxiety about scalpel, to control haemostasis, discomfort during procedure, and less scar formation [3, 5].

In the following case presentation, electrocautery has been used to perform the frenectomy. Electrocautery is a procedure that uses an electrical current to a metal wire or probe, which is, in turn, used to excise abnormal tissue. This method was used because it provides precise tissue-cutting, reduces bleeding, and minimizes the risk of infection and post-operative discomfort.

## Case presentation

**Figure 1:**
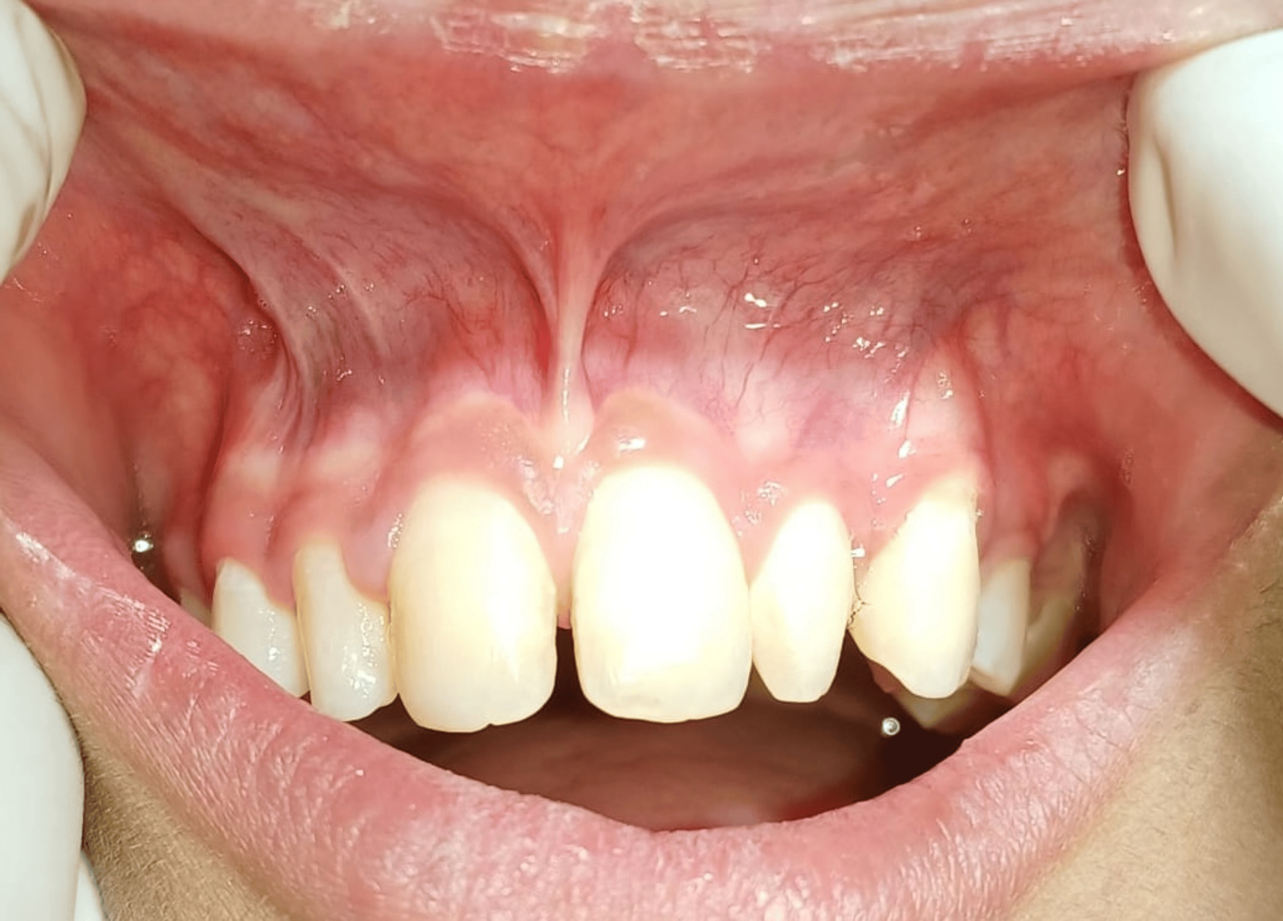
Pre-operative view showing midline diastema and abnormal frenum attachment which was of papillary type.

A 19-year-old female patient was referred from the Department of Orthodontics to the Department of Periodontics with the chief complaint of midline diastema and abnormal frenum attachment. The patient was diagnosed with a frenal attachment of papillary type. The patient had undergone orthodontic treatment one year ago and the relapse had occurred due to high frenal attachment (Figure 1).

**Figure 2:**
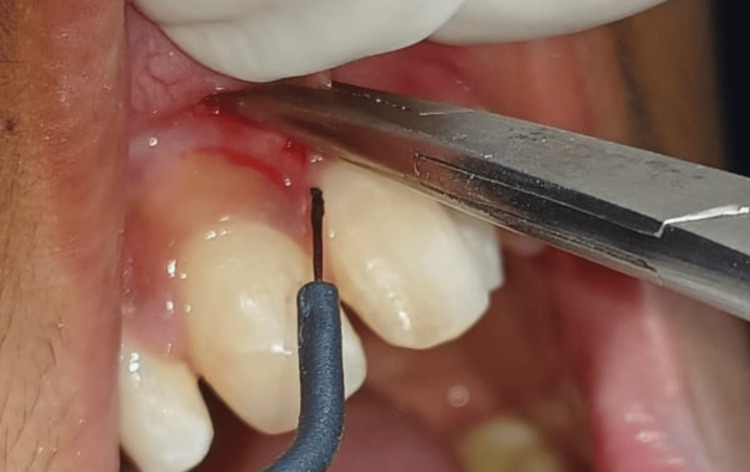
The haemostat was held engaging the abnormal frena and electrocautery was used to excise the frenum

The patient was advised frenectomy procedure. The patient was fearful about the blade and bleeding, therefore, the procedure was carried out with the electrocautery unit (Figure 2). Informed written consent was taken and haematological examinations were carried out, which were normal and within ranges.

**Figure 3:**
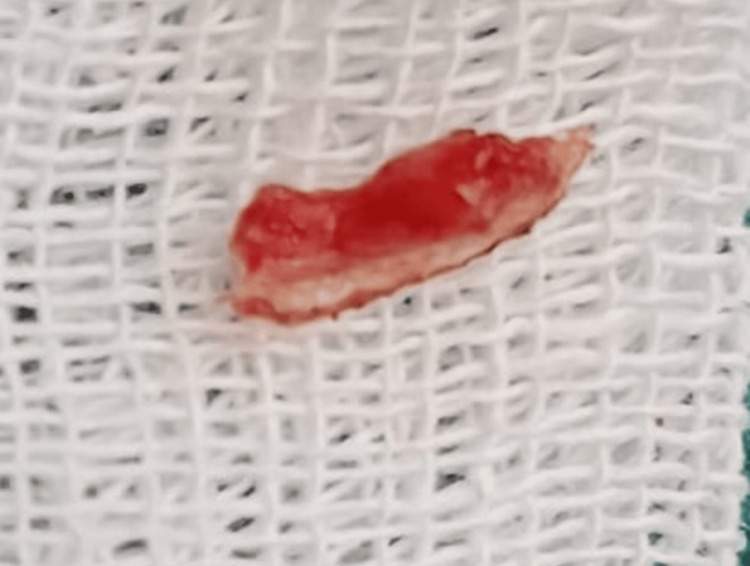
The upper and undersurface of frena was incised and a wedge-shaped triangular tissue was surgically excised by electrocautery

The surgical procedure was carried out using an electrocautery unit, where the haemostat is held between the frenum and the upper and undersurface triangular incision was given and wedge-shaped tissue was excised (Figure 3). 

**Figure 4:**
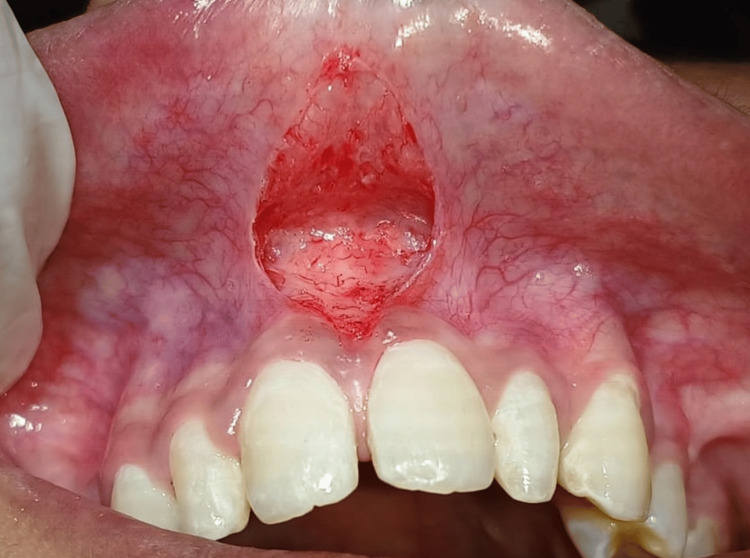
Post-operative picture showing diamond-shape incision after performing frenectomy with electrocautery where haemostasis achieved

Figure 4 shows a post-operative view after frenectomy where the bleeding did not occur and a diamond-shaped frenum incision line can be appreciated.

**Figure 5:**
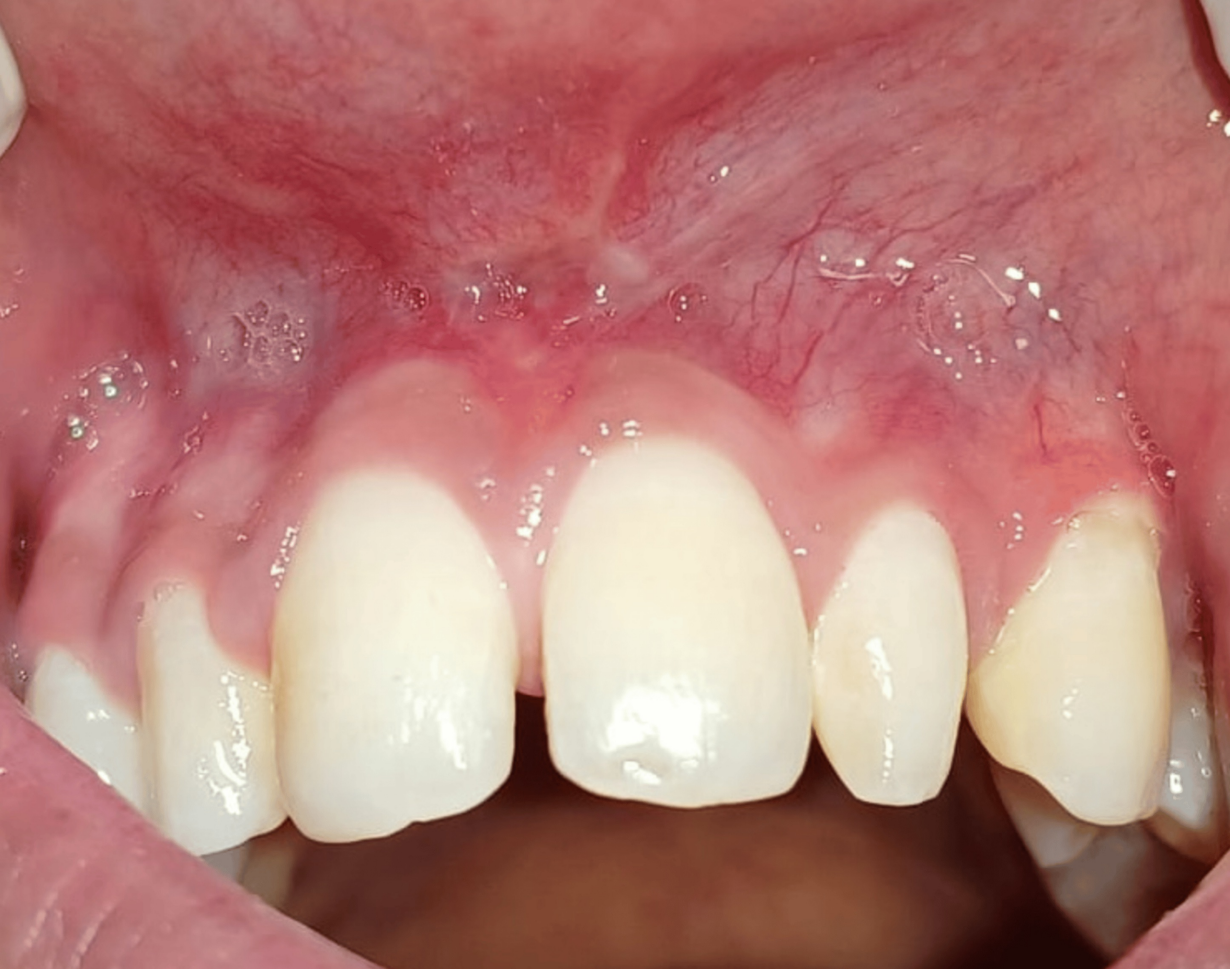
Post-operative view after 7 days showing satisfactory healing and no signs of infection

The patient was reviewed after 7 days and 3 months for periodic recall appointments. The post-operative picture in Figure 5 shows satisfactory healing, and the patient reported no discomfort and pain.

**Figure 6:**
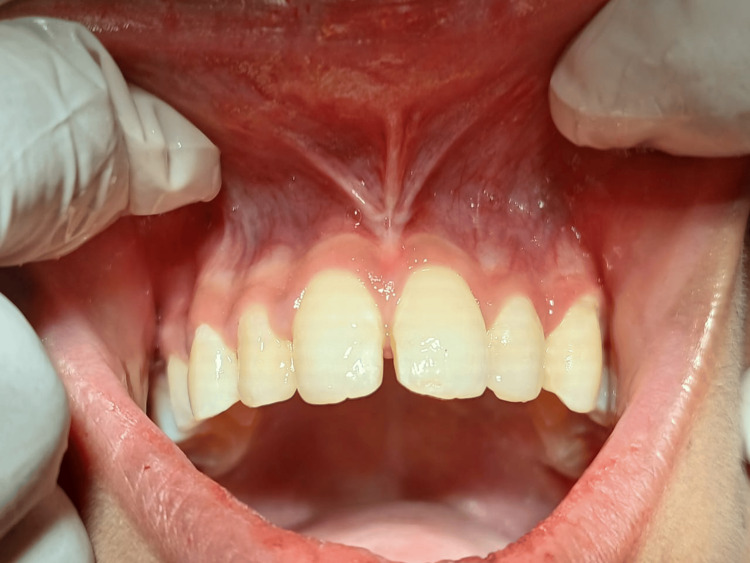
Post-operative view after 3 months showing complete and satisfactory healing and less scar formation

Figure 6 shows complete satisfactory healing, less scar formation and resulted in favourable outcomes for the treatment of midline diastema and mucogingival problems. 

## Discussion

The maxillary labial frenum is formed by the ectolabial bands that connect the palatine papilla to the tubercle of the upper lip as a post-eruptive remnant. It is considered pathogenic when the frenum is abnormally large or lacks a prominent attachment zone to the gingiva along the midline [4]. Despite the several modifications that have been suggested for frenectomy, a commonly employed approach that is still in use is the Classical technique, which was introduced by Archer (1961) and Kruger (1964) [6, 7]. However, there might be unaesthetic appearance and periodontal issues from this technique, which results in a scar and a longitudinal surgical incision.

Hence, modifications have been made to overcome such setbacks. To get around these complications, electrocautery was used in a case of the attached type of frenal attachment in a study by Devishree et al. [2] One advantage of the electrocautery approach is that the procedure can be performed in a bloodless field with less time, and no sutures are needed. Two main types of electrosurgical units used in dentistry are monopolar and bipolar. A bipolar unit is characterized by an arrangement in which two electrodes are positioned very close. An individual wire carries current from a monopolar device to the surgical site, and the device has just one electrode. For grounding, a pad must be placed on the patient's back. Hence, as the electrical current flows from one electrode to the other without a grounding pad, bipolar units are more expensive than diode lasers and monopolar units. As bipolar units have two wires, it results in a less precise cut when compared to its monopolar unit [8, 9].

In a study by Liboon et al., they compared mucosal incisions made by electrocautery, CO2 Laser, and scalpel. The electrosurgery unit demonstrated a faster rate of incisions and excisions in terms of seconds when compared to CO2 [10]. Electrocautery was used in conjunction with surgical excision of a pyogenic granuloma to maintain hemostasis. This method was shown to be straightforward, effective, and reliable in assisting bleeding and less scarring [11]. A strategically minimally invasive procedure for managing abnormal frenum is V-Y plasty in a study by Shirbhate et al., which provides the patient with cosmetic benefits and minimally scarred aberrant high-maxillary frenal attachments [12]. Because electrocautery is safe and effective, it is advised for frenectomy procedures. It's a treatment with minimal bleeding and no problems following surgery [13].

## Conclusions

The frenectomy procedure was done by using electrocautery to remove the high labial frenum attachment. The case demonstrates uneventful post-operative healing and no development of hypertrophic scars, which met the surgeon’s and the patient’s functional and esthetic demands. The treatment showed complete and satisfactory healing and achieved the desired, favourable outcomes. Electrocautery effectively provided both functional and esthetic improvements in the context of midline diastema even after orthodontic treatment.
